# Identification and validation of aging-related genes in heart failure based on multiple machine learning algorithms

**DOI:** 10.3389/fimmu.2024.1367235

**Published:** 2024-04-15

**Authors:** Yiding Yu, Lin Wang, Wangjun Hou, Yitao Xue, Xiujuan Liu, Yan Li

**Affiliations:** ^1^ Shandong University of Traditional Chinese Medicine, Jinan, China; ^2^ Affiliated Hospital of Shandong University of Traditional Chinese Medicine, Jinan, China

**Keywords:** heart failure, aging, machine learning, bioinformatics, immune infiltration analysis

## Abstract

**Background:**

In the face of continued growth in the elderly population, the need to understand and combat age-related cardiac decline becomes even more urgent, requiring us to uncover new pathological and cardioprotective pathways.

**Methods:**

We obtained the aging-related genes of heart failure through WGCNA and CellAge database. We elucidated the biological functions and signaling pathways involved in heart failure and aging through GO and KEGG enrichment analysis. We used three machine learning algorithms: LASSO, RF and SVM-RFE to further screen the aging-related genes of heart failure, and fitted and verified them through a variety of machine learning algorithms. We searched for drugs to treat age-related heart failure through the DSigDB database. Finally, We use CIBERSORT to complete immune infiltration analysis of aging samples.

**Results:**

We obtained 57 up-regulated and 195 down-regulated aging-related genes in heart failure through WGCNA and CellAge databases. GO and KEGG enrichment analysis showed that aging-related genes are mainly involved in mechanisms such as Cellular senescence and Cell cycle. We further screened aging-related genes through machine learning and obtained 14 key genes. We verified the results on the test set and 2 external validation sets using 15 machine learning algorithm models and 207 combinations, and the highest accuracy was 0.911. Through screening of the DSigDB database, we believe that rimonabant and lovastatin have the potential to delay aging and protect the heart. The results of immune infiltration analysis showed that there were significant differences between Macrophages M2 and T cells CD8 in aging myocardium.

**Conclusion:**

We identified aging signature genes and potential therapeutic drugs for heart failure through bioinformatics and multiple machine learning algorithms, providing new ideas for studying the mechanism and treatment of age-related cardiac decline.

## Introduction

With an aging global population and improved survival rates for ischemic heart disease due to increasingly effective, evidence-based treatments, heart failure prevalence is on the rise, now affecting over 64 million individuals worldwide (1). This trend is particularly pronounced among elderly patients. As such, the escalating elderly demographic intensifies the urgency to both comprehend and counteract age-related cardiac deterioration. This necessitates the exploration of novel pathological and cardioprotective mechanisms, aiming to reduce the extensive impact on global public health (2).

Heart failure’s development is intricately linked to the complex interplay of cardiovascular aging, risk factors, comorbidities, and disease moderators (3). While dietary restrictions, increased physical activity, and pharmacological interventions are pivotal in decelerating cardiovascular function decline in aging populations, their impact on mortality remains limited (4, 5). Recent research posits that the persistent high prevalence of cardiovascular diseases and associated mortality may stem from a lack of targeted interventions addressing the aging process directly. These studies have identified eight key molecular markers characteristic of cardiovascular aging: impaired macroautophagy, proteostasis loss, genomic instability, epigenetic changes, mitochondrial dysfunction, cellular senescence, disrupted neurohormonal signaling, and inflammation (6).

With advancing age, significant structural and functional transformations occur in the heart, blood vessels, and microcirculation (7). These changes in cardiac structure and function contribute to an increased vulnerability to heart failure in the elderly. However, the precise mechanisms by which aging precipitates heart failure are not yet fully understood. Unraveling the specific genes and molecular processes involved in the onset and progression of heart failure during aging is crucial. Such insights are expected to pave the way for innovative strategies to combat age-related decline, preserve circulatory function, and extend the disease-free lifespan of individuals.

Bioinformatics, an ever-evolving multidisciplinary domain, is revolutionizing our understanding in the medical sciences. This study leverages high-throughput technologies and machine learning to unearth pivotal aging genes and molecular pathways implicated in heart failure. Machine learning is currently applied to the further screening of key genes, providing more precise results compared to traditional PPI network screening (8). This is because machine learning methods can identify more complex nonlinear relationships. LASSO, a regression-based method, can perform feature selection by shrinking the coefficients of less important features to zero, making it highly suitable for high-dimensional data. However, LASSO’s feature selection can be unstable (9). By recursively removing features with the smallest weights, SVM-RFE can effectively perform feature selection and handle linearly inseparable data. Yet, the choice of kernel function and parameter tuning significantly affects SVM’s performance (10). The RF algorithm, an ensemble learning method comprising multiple decision trees, handles nonlinear data effectively and reduces the risk of overfitting by establishing multiple decision trees. However, RF models are usually hard to interpret (11). Therefore, we tend to combine the results of these three algorithms to enhance the model’s accuracy.

**Figure 1:**
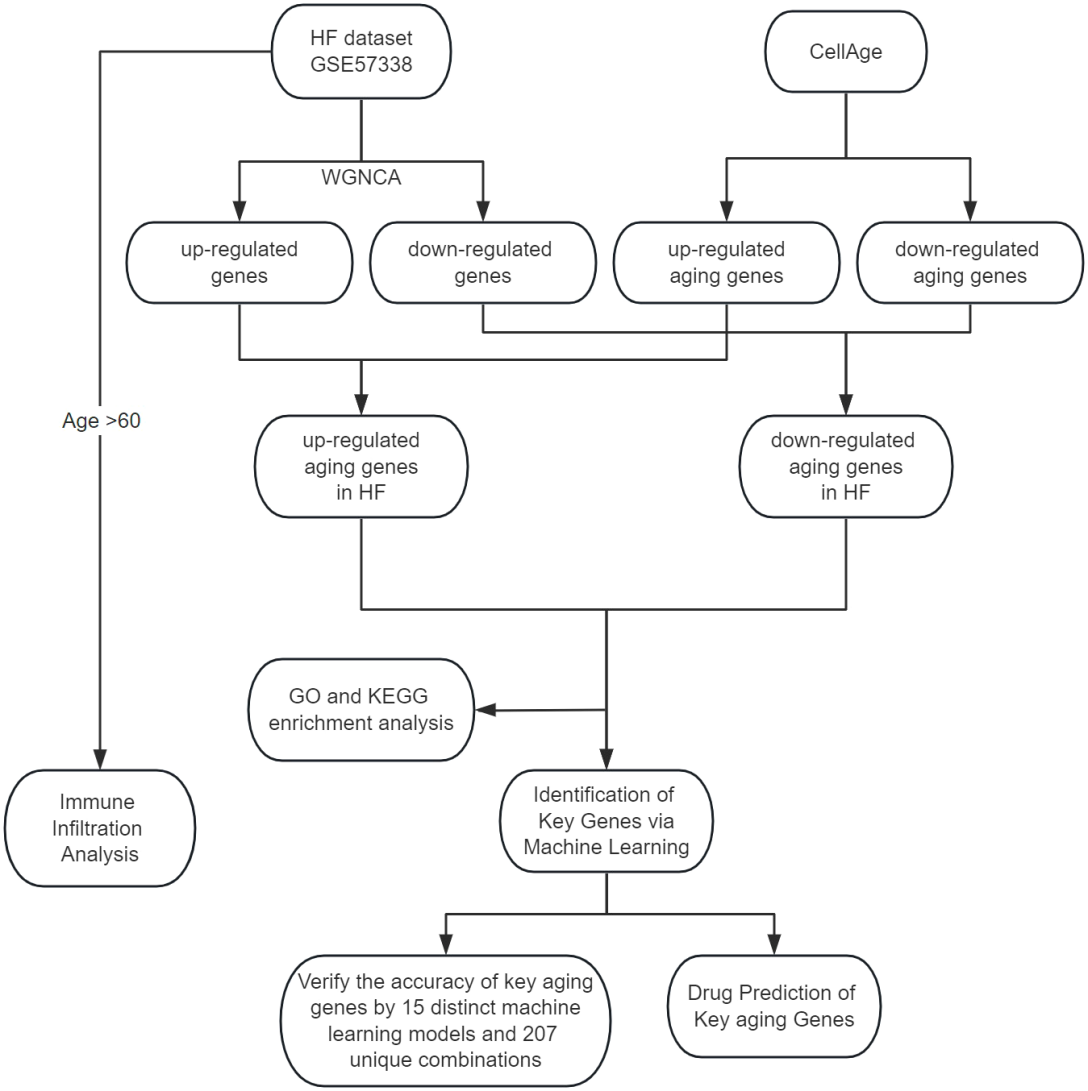
The study flowchart.

Utilizing Weighted Gene Co-expression Network Analysis (WGCNA), we identified crucial module genes from the largest available heart failure dataset, integrating these findings with the CellAge database to highlight aging-related genes. Subsequent feature enrichment analysis led to the selection of three advanced machine learning algorithms: Least Absolute Shrinkage and Selection Operator (LASSO), Random Forest (RF), and Support Vector Machine Recursive Feature Elimination (SVM-RFE), for pinpointing key genes associated with heart failure and aging. To ascertain the robustness of our findings, we employed a comprehensive validation approach, testing the results across a primary test set and two external datasets using 15 distinct machine learning models and 207 unique combinations. We conducted a comprehensive drug prediction analysis utilizing the Drug Signatures Database (DSigDB). This approach was instrumental in identifying potential pharmaceutical candidates for the management and treatment of heart failure and associated aging processes. In the final phase of our study, we used CIBERSORT to assess the content of immune cells and stromal cells in aged myocardium to delineate the cellular heterogeneity landscape of expression profiles in aged myocardium. The methodology and progression of this study are encapsulated in **Figure 1**, which outlines the research flowchart.

## Materials and methods

### Data acquisition and preprocessing

This study’s data were sourced from the publicly accessible Gene Expression Omnibus (GEO) database, with the datasets having previously obtained participant consent and ethical approval (12). Consequently, our research did not require additional approval from an institutional review board. We selected GSE57338 as our primary dataset for ischemic heart failure analysis due to its extensive sample size, comprising left ventricular myocardial samples from 95 ischemic heart failure patients and 136 individuals without heart failure (13). GSE57338 documents data for over 20,000 mRNAs from the left ventricular myocardium across 231 samples. In terms of age composition, 86 samples are from individuals aged 60 and above. Regarding gender composition, 154 samples come from males. For external validation, we utilized datasets GSE5406 (including myocardial samples from 108 ischemic heart failure patients and 16 non-heart failure individuals) and GSE16499 (comprising samples from 15 ischemic heart failure patients and 15 non-heart failure individuals) (14, 15).

Data preprocessing was conducted using R software (version 4.2.0). In this process, we eliminated probes linked to multiple molecules. Where multiple probes corresponded to a single molecule, only the probe with the highest signal value was retained. To ensure data consistency and accuracy, we also corrected for batch effects in the data and converted probe IDs to gene symbols based on the platform’s annotation file.

### Weighted gene co-expression network analysis

We used the WGCNA package to explore gene modules associated with heart failure (The samples consisted of 136 normal samples and 95 ischemic heart failure samples from GSE57338.) (16). WGCNA can identify clusters of highly correlated genes, summarizing these clusters using either the module eigengene or an intramodular hub gene, and relate the modules to each other and to external sample traits. Within these modules, WGCNA is capable of identifying key driver genes or central genes that play critical roles in disease processes, as these genes exhibit the highest connectivity within the module and are positioned most centrally within the network. Using 0.5 as the filtering standard and removing unqualified genes and samples through the goodSamplesGenes function, a scale-free co-expression network was established. Subsequently, adjacency was calculated with a default soft threshold of β = 30 and scale-free R2 = 0.9, and the adjacency was converted into a topological overlap matrix (TOM) to determine gene ratios and dissimilarity. Genes with the same expression profile are divided into gene modules using average linkage hierarchical clustering, we prefer larger modules, so we set the minimum module size to 300. Finally, the dissimilarity of module characteristic genes is calculated, the cutting line of the module dendrogram is selected to combine several modules for further research, and the visualization of the characteristic gene network is completed.

### Screening candidate aging-related genes in HF

The CellAge database (https://genomics.senescence.info/cells/) serves as a comprehensive repository of human genes associated with cellular senescence (17). This database meticulously catalogs genes with established positive, negative, or undetermined impacts on this process. We involved correlating genes upregulated in heart failure (HF) with those in CellAge known to accelerate cell senescence. Concurrently, we analyzed the overlap of genes downregulated in HF with those identified in CellAge as senescence inhibitors. This dual-faceted approach facilitated the identification of key aging-related genes specifically involved in the pathophysiology of heart failure.

### Functional enrichment analysis

To elucidate the biological processes and functions of aging genes implicated in heart failure, our study utilized the clusterProfiler package (18). This tool enabled us to conduct a comprehensive Gene Ontology (GO) and Kyoto Encyclopedia of Genes and Genomes (KEGG) enrichment analysis (19, 20). Through this analysis, we were able to identify and visualize key pathways and gene functions, providing deeper insights into how aging genes contribute to the pathophysiology of heart failure.

### Machine learning

In our investigation, we employed three distinct machine learning algorithms—LASSO, RF, and SVM-RFE—to rigorously identify key aging genes in HF (21–23). The LASSO algorithm was executed using the glmnet package, incorporating ten-fold cross-validation to pinpoint significant genes. For the RF algorithm, we utilized the randomForest package, selecting the top 20 genes as our primary candidates. The SVM-RFE algorithm, conducted via the e1071 package, was used to determine the optimal gene subset based on accuracy. The culmination of these methodologies was the identification of a consensus set of genes, representing the intersection of results from all three algorithms, which we designated as the critical aging genes in heart failure.

### Validation of key aging genes

In order to verify the accuracy of key aging genes, we integrated 15 machine learning algorithms (included Neural Networks, Logistic Regression, Linear Discriminant Analysis, Quadratic Discriminant Analysis, K-Nearest Neighbors, Decision Trees, Random Forest, XGBoost, Ridge Regression, LASSO Regression, Elastic Net Regression, Support Vector Machines, Gradient Boosting Machines, Stepwise Logistic Regression, and Naive Bayes) and combined these 15 algorithms through caret parameter adjustment, custom parameter combination, lasso feature screening, and cross-validation, resulting in a total of 207 machines learning model. For our analysis, we randomly allocated 70% of the GSE57338 dataset as the training set and designated the remaining 30% for testing. In addition, we incorporated two external validation sets, GSE5406 and GSE16499, to further strengthen the robustness of our results.

### Drug prediction

DSigDB, a comprehensive drug signature database, was employed for gene set analysis in our study (24). We utilized the identified key aging genes as a reference list, applying DSigDB’s predictive capabilities to identify potential drug molecules.

### Immune infiltration analysis

Aging is often accompanied by a decline in immune function. Therefore, we used CIBERSORT to evaluate the content of immune cells and stromal cells in aged HF myocardial samples to delineate the cellular heterogeneity landscape of aging myocardial expression profiles and complete immune cell infiltration analysis (25). The samples were derived from patients older than 60 years old in GSE57338. To assess whether the levels of immune cells in patients’ circulation are correlated with myocardial immune levels, we also conducted an immune infiltration analysis on GSE77343(GSE77343 records whole blood samples from 197 heart failure patients). Bar charts are used to visualize the proportion of each type of immune cell in different samples. The differences in cell distribution between the HF group and the normal group were compared by t test, and the cutoff value was set at p<0.05.

## Results

### Construction of co-expressed gene modules

**Figure 2:**
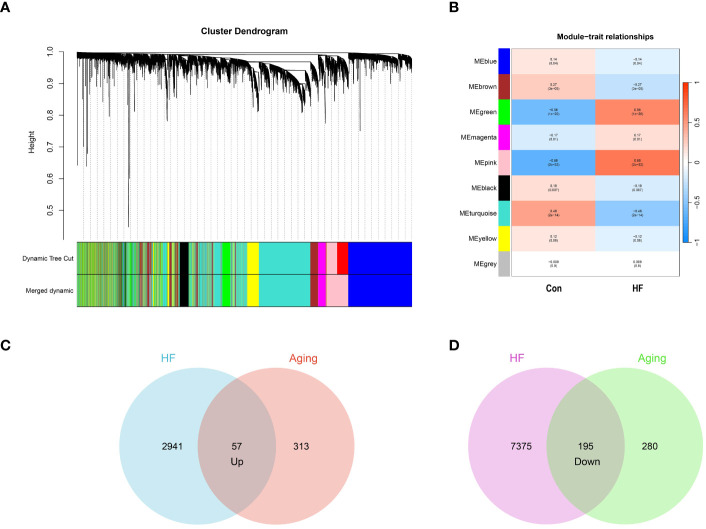
Identification of aging-related genes. **(A)** Gene and trait clustering dendrograms of HF. Gene clustering trees (dendrograms) obtained by hierarchical clustering of neighbor-based differences. **(B)** 9 gene co-expression modules of HF. The numbers in each cell means the correlation coefficient and p-value. **(C)** 57 genes promote both aging and HF. **(D)** 195 genes inhibit both aging and HF.

In our study, we conducted WGCNA on the GSE57338 dataset, identifying both upregulated and downregulated modules significantly associated with HF. The analysis revealed that a β value of 10 brought the network closest to a scale-free topology. Within this framework, we pinpointed 9 modules related to HF. Notably, in the upregulated category, the pink module (correlation coefficient = 0.68, P = 2e-32) and the green module (correlation coefficient = 0.56, P = 1e-20) demonstrated the highest correlation with HF, encompassing a combined total of 2,998 genes. Conversely, among the downregulated modules, the turquoise module exhibited the strongest association with HF (correlation coefficient = 0.48, p = 2e-14), comprising 7,570 genes. These findings are represented in **Figures 2A** and **2B**.

From the CellAge database, we identified 370 genes implicated in promoting aging and 475 genes associated with inhibiting aging. We then conducted an intersection analysis between these aging-related genes and those influencing heart failure. This approach revealed 57 genes that concurrently promote both aging and heart failure. We also identified 195 genes that play a role in inhibiting both aging and heart failure. The results of these intersection analyses are represented through Venn diagrams in **Figures 2C** and **2D**. The genes associated with WGCNA modules and CellAge can be found in **Supplementary S1**.

### Functional enrichment analysis

We performed GO and KEGG enrichment analysis on 252 aging-related genes in heart failure. This was undertaken to elucidate the shared biological mechanisms underpinning both conditions. The GO enrichment analysis encompassed three primary categories: Biological Process (BP), Cellular Component (CC), and Molecular Function (MF). Notably, BP categories were predominantly focused on aspects like histone modification, regulation of the mitotic cell cycle, cell cycle phase transition, and positive regulation of cell division. CC categories emphasized elements such as focal adhesion, cell-substrate junctions, pericentric heterochromatin, SWI/SNF superfamily-type complexes, and ATPase complexes. In the MF category, significant functions included histone binding, DNA-binding transcription factor interaction, transcription coregulator and corepressor activities, and NAD-dependent histone deacetylase activity.

**Figure 3:**
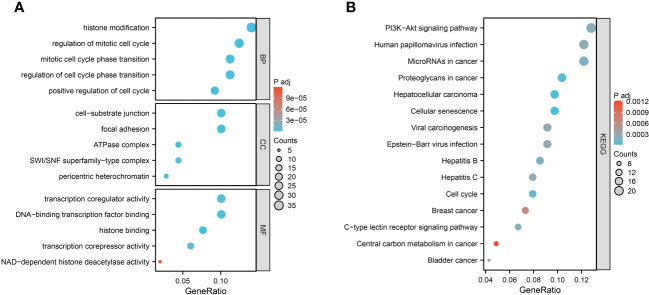
Function enrichment analysis of 252 aging-related genes. **(A)** GO enrichment analysis results. **(B)** KEGG enrichment analysis results.

The KEGG enrichment analysis revealed that these aging-related genes in heart failure were significantly enriched in pathways including Cellular Senescence, Proteoglycans in Cancer, Cell Cycle, MicroRNAs in Cancer, C-type Lectin Receptor Signaling Pathway, PI3K-Akt Signaling Pathway, and various cancer-related signaling pathways. The top five results from the GO enrichment analysis and the top ten from the KEGG enrichment analysis will be presented, as depicted in **Figure 3**.

### Identification of key genes via machine learning

We used three machine learning algorithms, LASSO, RF and SVM-RFE, to further screen key aging genes in heart failure. Among the up-regulated genes, the LASSO algorithm identified 17 candidate genes. The RF algorithm ranks genes according to the importance calculation of each gene, and we select the top 20 genes as candidate genes. The SVM-RFE algorithm shows that the accuracy is highest when 34 genes are included, so we selected the first 34 genes of the SVM-RFE algorithm as candidate genes. After intersecting the results of the three algorithms, we obtained 10 up-regulated key aging genes in heart failure, namely CDKN1B, SPIN1, GNMT, HTRA1, ITPK1, MAVS, MME, RAF1, TLR3, and XAF1.

**Figure 4:**
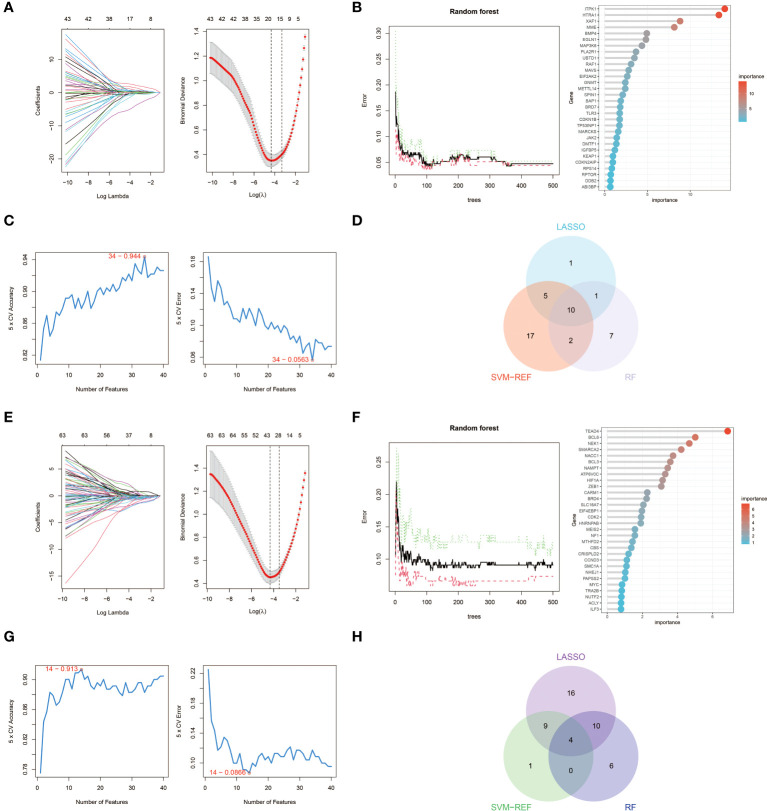
Machine learning in screening key aging genes for HF. **(A)** Screening of key aging genes using the Lasso Model in up-regulated genes. The Lasso coefficient profiles were utilized to identify the optimal feature genes, with the optimal lambda determined by minimizing the partial likelihood deviance. Each coefficient curve in the left picture represents an individual gene. The solid vertical lines in the right picture represent the partial likelihood deviance, and the number of genes (n = 17) corresponding to the lowest point of the curve was deemed most suitable for the Lasso model. **(B)** Screening of key aging genes using the RF Model in up-regulated genes. The relative importance of overlapping candidate genes was calculated using the random forest approach. We present the results for the top 20 genes. **(C)** Screening of key aging genes using the SVM-RFE Model in up-regulated genes. The SVM-RFE algorithm was employed to further identify the optimal feature genes, based on the highest accuracy and lowest error obtained from the curves. The x-axis indicates the number of feature selections, while the y-axis represents the prediction accuracy. **(D)** Venn diagram illustrating the identification of 10 candidate genes for up-regulated genes through the aforementioned three algorithms. **(E)** Screening of key aging genes using the Lasso Model in down-regulated genes. **(F)** Screening of key aging genes using the RF Model in down-regulated genes. **(G)** Screening of key aging genes using the SVM-RFE Model in down-regulated genes. **(H)** Venn diagram shows that 4 key aging genes for down-regulated genes are identified via the above three algorithms.

Similarly, among the down-regulated genes, the LASSO algorithm identified 39 candidate genes. We still select the top 20 genes of the RF algorithm as candidate genes. The SVM-RFE algorithm shows that the accuracy is highest when 14 genes are included, so we selected the first 14 genes of the SVM-RFE algorithm as candidate genes. After intersecting the results of the three algorithms, we obtained four down-regulated key aging genes in heart failure, namely BCL6, EIF4EBP1, MEIS2, and SMARCA2. The visualization results are shown in **Figure 4**. The results from each machine learning algorithm and the lists of the key genes can be found in **Supplementary S2**.

### Key genes verification

**Figure 5:**
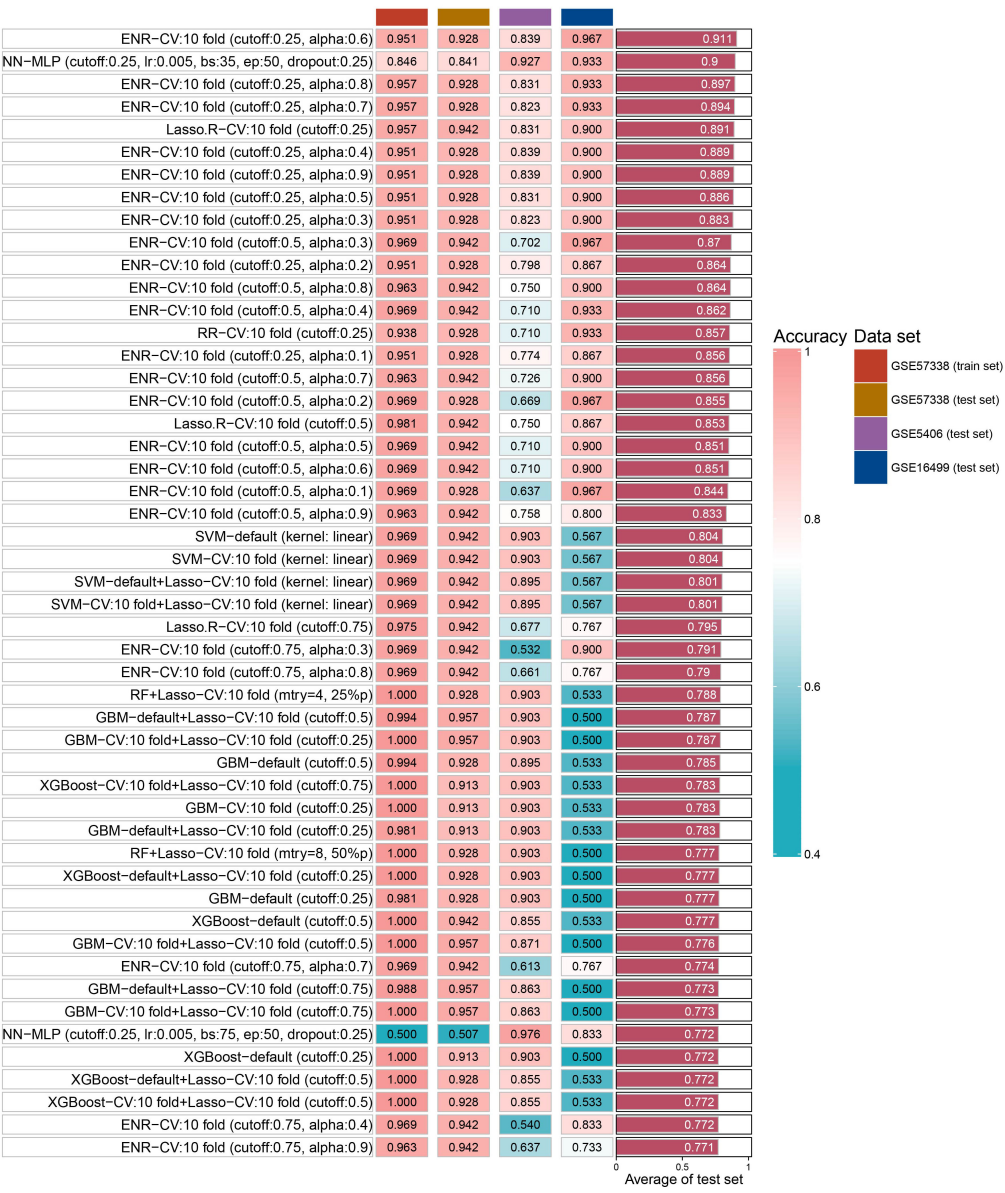
The accuracy of top 50 machine- learning algorithm combinations.

We identified 14 key aging genes associated with heart failure using three distinct machine learning algorithms. To circumvent the limitations imposed by the sample size in the ROC curve analysis of combined genes, we pursued an alternative validation strategy. Specifically, we validated these 14 genes using 15 different machine learning algorithm models and 207 combinations, across both the test set and two external validation sets. The validation results indicated that, in most algorithms, the accuracy of these 14 genes exceeded 0.8 in both the test set and external validation sets. Notably, the Elastic Net Regularized Generalized Linear Model with Cross-Validation (ENR-CV), with specific parameters set to 10-fold cross-validation, a cutoff value of 0.25, and an alpha value of 0.6, achieved the highest average accuracy (0.911). The top 50 average accuracy rankings from this comprehensive analysis are depicted in **Figure 5**.

### Drug prediction of key genes

In our pursuit to identify potential pharmacological agents for tackling heart failure in aging patients, we utilized the DSigDB database. Our selection criteria focused on drugs with an Adjusted P-value of less than 0.01. This stringent threshold led to the identification of six promising drug candidates: Arsenenous acid, Cyclophosphamide, Lovastatin, Rimonabant Hydrochloride, Sorafenib, and Alvespimycin. Detailed information about these drugs was provided in **Table 1**.

**Table 1:** The details of drugs.

Drugs	Combined Score	Genes	Molecular Formula
Arsenenous acid	168.64	CDKN1B;BCL6;HTRA1;EIF4EBP1;RAF1;XAF1;TLR3	AsHO_2_
Cyclophosphamide	294.17	CDKN1B;BCL6;EIF4EBP1;XAF1	C_7_H_15_Cl_2_N_2_O_2_P
Lovastatin	586.32	CDKN1B;RAF1;SMARCA2	C_24_H_36_O_5_
Rimonabant hydrochloride	2335.08	CDKN1B;RAF1	C_22_H_22_Cl_4_N_4_O
Sorafenib	478.17	CDKN1B;EIF4EBP1;RAF1	C_21_H_16_ClF_3_N_4_O_3_
Alvespimycin	1992.62	CDKN1B;RAF1	C_32_H_48_N_4_O_8_

### Immune infiltration analysis of aging myocardium

**Figure 6:**
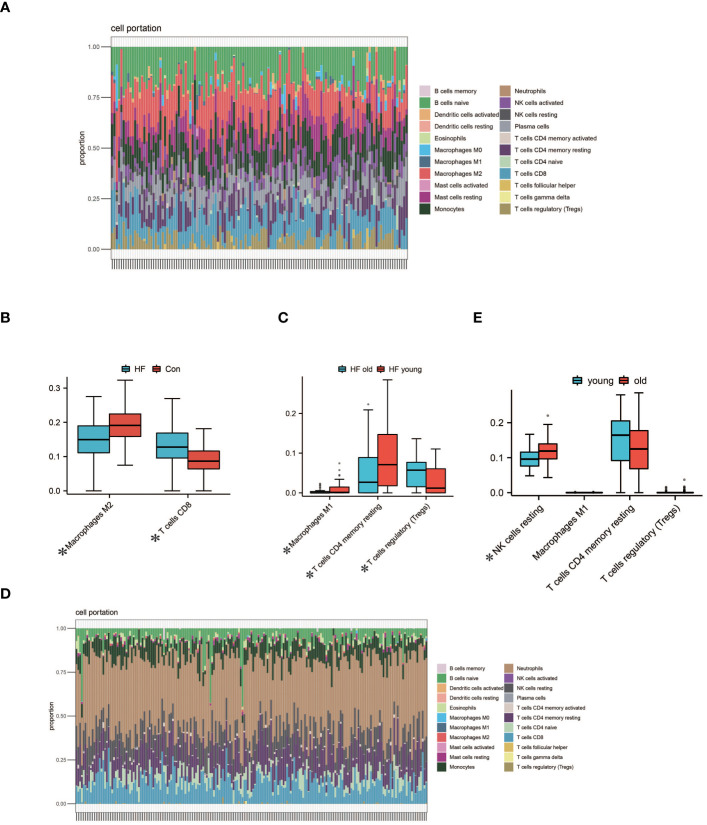
Analysis of Immune Cell Infiltration. **(A)** Visualization from bar graphs of the proportions of 22 types of immune cells in elderly healthy samples, elderly heart failure samples, and young heart failure samples from GSE57338. **(B)** Expression of 2 dysregulated immune cells in elderly heart failure samples and elderly healthy samples (*indicates p<0.05, the same below). **(C)** Expression of 3 dysregulated immune cells in elderly heart failure samples and young heart failure samples. **(D)** Visualization of bar graphs showing the proportions of 22 types of immune cells at the circulating level in elderly heart failure samples and young heart failure samples from GSE77343. **(E)** The proportion of 1 dysregulated immune cell in elderly heart failure samples and young heart failure samples. This indicates differences between myocardial immune cell levels and circulating immune cell levels.

We performed immune infiltration analysis on myocardial samples from 56 elderly heart failure patients and 32 normal elderly patients in GSE57338 using the CIBERSORT algorithm. The bar graph clearly shows the different subpopulation content in each sample. We evaluated cellular composition heterogeneity between elderly heart failure samples and elderly healthy samples, and the results showed that 2 types of immune cell infiltration were significantly different. Macrophages M2 of normal samples were lower than those of heart failure samples, and T cells CD8 were higher than those of heart failure samples. The differences of these two types of immune cells may provide potential regulatory points for the treatment of heart failure in the elderly. In myocardial samples from both young and elderly heart failure patients, three types of dysregulated immune cells were identified: Macrophages M1, T cells CD4 memory resting, and T cells regulatory. However, analysis of whole blood samples revealed that these three types of immune cells were not dysregulated in circulation; instead, resting NK cells were found to be dysregulated. The visualization results are shown in **Figure 6**. The statistical analysis results can be found in detail in **Supplementary S3**.

## Discussion

Heart failure is a disease caused by structural changes or functional impairment of the heart, and aging plays an important role in its progression (26). In fact, among the elderly, maintaining normal circulatory function helps increase disease-free life expectancy and maintain a higher quality of life (27). As the aging of the population increases and the survival rate of ischemic cardiomyopathy increases, heart failure in the elderly population has brought serious economic and public health burdens (28). Although the common mechanisms of heart failure and aging are an active research topic, current research focus is still on investigating the potential anti-aging mechanisms of heart failure therapeutic drugs, and the aging-related mechanisms and potential therapeutic drugs for heart failure remain unclear (29). Therefore, the purpose of this study was to identify and verify aging-related genes in heart failure, and to explore the mechanism and potential therapeutic drugs of aging in heart failure, in order to reveal new pathological mechanisms and cardioprotective pathways.

In this study, we first obtained 252 aging-related genes in heart failure through WGCNA and CellAge databases, and explored the biological functions and signaling pathways involved in aging genes through GO and KEGG enrichment analysis. The results show that biological processes are mainly related to histone modifications and cell cycle. Histone modifications are chemical modifications of histone amino acid residues, which can regulate gene expression without changing the DNA sequence, including methylation, acetylation, ubiquitination, etc (30). Histone modifications are dynamically regulated under cardiac stress, leading to heart failure through compensatory or maladaptive transcriptome reprogramming (31). Studies have shown that histone acetylation regulators can affect processes such as cardiomyocyte hypertrophy, apoptosis, fibrosis, oxidative stress, and inflammation, and exert cardioprotective effects (32). Regulation of histone methylation and acetylation modifiers serves as a bridge between signaling and downstream gene reprogramming, and regulation of their levels helps define the epigenetic landscape required for correct cardiomyocyte function (33). However, in aging individuals, due to histone loss, abnormal modifications, and accumulation of mutations, the strict regulation of histone modifications begins to disintegrate, disrupting tissue homeostasis and regeneration (34). Given the reversibility of epigenetic regulation, epigenetic modifiers hold exciting promise in both delaying aging and treating heart failure (35). Loss of cardiac contractile substrate and limited myocyte regenerative capacity are major contributors to poor outcomes in heart failure (36). The heart is an organ with poor regenerative capacity, and it is difficult for cardiomyocytes to re-enter the cell cycle for regeneration and repair. Studies have shown that a combination of cell cycle regulators can induce stable cytokinesis in adult postmitotic cells and significantly improve cardiac function after acute or subacute myocardial infarction (37). Additionally, forcing cardiomyocytes to proliferate while minimizing the oncogenic potential of cell cycle factors using novel transient and cardiomyocyte-specific viral constructs may reduce arrhythmias or systemic tumorigenesis while sustainably improving cardiac function (38). Aging requires cell cycle arrest in response to damaging stimuli, and therefore, cell cycle modulators may have better efficacy in treating heart failure in the aging population (39).

KEGG enrichment results show that the aging genes in heart failure mainly involve cellular senescence, proteoglycans in cancer, cell cycle, microRNAs in cancer, c-type lectin receptor signaling pathway, PI3K-Akt signaling pathway, and signal pathways related to cancer diseases. Cellular aging, characterized by an irreversible arrest in the cell cycle induced by stress, markedly impairs various cellular functions, including homing, proliferation, migration, and differentiation (40). Beyond the hallmarks of DNA damage, endoplasmic reticulum stress, and mitochondrial dysfunction, senescent cardiomyocytes also exhibit an age-related secretory phenotype. This phenotype involves the release of pro-inflammatory cytokines, chemokines, and matrix-degrading enzymes, which detrimentally influence the myocardial microenvironment and neighboring healthy cardiomyocytes, exacerbating cardiac remodeling and failure (41). Therefore, mitigating the decline in cardiac function in aging organisms necessitates not only the activation of maintenance and repair mechanisms but also prioritizing the induction of apoptosis in senescent cells, a strategy that holds promise as a therapeutic approach (42, 43). Senescent cells frequently exhibit activation of the PI3K-Akt signaling pathway, a phenomenon not observed in younger cells (44). Interestingly, reducing AKT and ERK activation has proven effective in extending lifespan in Drosophila (45). However, this poses a paradox, as the amelioration of myocardial fibrosis and protection of cardiac cells often entail activating the PI3K-Akt pathway (46–48). Thus, the challenge lies in striking a balance between mitigating heart failure and aging when modulating the PI3K-Akt signaling pathway, a key area for future research. Heart failure-associated aging genes have been found to be significantly enriched in pathways commonly implicated in cancer. This correlation may stem from the intricate relationship between cellular aging, the cell cycle, and cancer. While aging naturally serves as a deterrent against tumorigenesis, senescent cells, both malignant and non-malignant, under certain conditions, can paradoxically adopt tumor-promoting characteristics (49, 50). Consequently, therapies that promote aging processes present as a viable strategy in cancer treatment (51). Nonetheless, the multifaceted role of aging in diverse physiological and pathological contexts necessitates a careful consideration of the cardiac implications of pro-aging therapies in cancer patients. Furthermore, the role of cell division in cancer progression is critical; inaccuracies during this process can lead to chromosomal content variations and aneuploidy, thereby contributing to oncogenesis (52). Research indicates a reduction in cell proliferation within the aging transcriptome, contrasted by a shift towards heightened cell division in the cancer transcriptome (53). This observation suggests that a strategic, sequential application of pro-aging therapy followed by anti-aging treatment may offer a balanced approach, mitigating organ-specific burdens in cancer patients (54).

In order to explore the key aging genes in heart failure, we used three machine learning algorithms to obtain 10 key up-regulated genes and 4 key down-regulated genes. Subsequently, we fit the 14 key genes on 15 machine learning algorithm models and 207 combinations and validated them in two independent external data sets. The results showed that the best average accuracy was 0.911, which shows that these 14 key genes can be used as aging signature genes for heart failure. This discovery paves the way for further exploration of crucial aging-related mechanisms in heart failure and the development of targeted therapeutics. Notably, the reproducibility of our findings was corroborated by their consistency across two separate and independent external datasets.

Our study identified 10 key up-regulated genes predominantly involved in cell cycle regulation, programmed cell death, and immune response. Among these, CDKN1B and SPN1 emerge as vital regulators of cell cycle progression. CDKN1B acts as a principal driver of cell division and plays a crucial role in restraining abnormal cell proliferation (55). SPN1, associated with meiotic spindles, has been observed to induce metaphase arrest and chromosomal instability upon overexpression (56).In the realm of programmed cell death, genes like ITPK1, MAVS, RAF1, and XAF1 play diverse roles. ITPK1 intervenes in TNF-α-induced apoptosis by disrupting the activation of the TNFRSF1A-associated death domain and is implicated in the oligomerization and localization of activated pMLKL to the cell membrane, thereby modulating necroptosis (57, 58). MAVS, while offering apoptosis resistance, also mediates the recruitment of NLRP3 to mitochondria, triggering the activation of the NLRP3 inflammasome and consequent pyroptosis (59, 60). RAF1 acts as a critical link within the MAPK/ERK cascade, determining cell fate across a spectrum of processes such as growth, proliferation, migration, differentiation, and survival (61). It also safeguards cells from apoptosis through NF-kappa B activation and its mitochondrial translocation to bind with BCL2 (62). XAF1, in synergy with TNF-α, induces apoptosis and is involved in trophoblast cell apoptosis (63).Furthermore, TLR3 plays a pivotal role in both innate and adaptive immunity. It operates via the TRIF/TICAM1 adapter, leading to NF-kappa B activation, IRF3 nuclear translocation, cytokine secretion, and inflammatory responses (64).

The 4 key genes identified as down-regulated in our study play diverse roles in various biological processes. BCL6 functions as a transcriptional repressor, primarily in germinal center B cells, where it inhibits genes associated with differentiation, inflammation, apoptosis, and cell cycle regulation (65). MEIS2, known for promoting the proliferation of cardiac myoblasts, exhibits decreased expression in aging individuals, potentially exacerbating the decline in cardiac function (66). SMARCA2 is implicated in transcriptional activation and selective gene repression via chromatin remodeling. Research indicates that the SWI/SNF ATP-dependent chromatin remodeling complex is vital for maintaining metabolic homeostasis in adult cardiomyocytes (67). Lastly, EIF4EBP1, which is phosphorylated in response to signals such as insulin, plays a role in the regulation of mRNA translation upon dissociation from eIF4E. This gene is also implicated in processes like autophagy and acts as a crucial effector in the mTOR signaling pathway (68, 69).

After obtaining the key aging genes of heart failure, we tried to search for potential drug molecules that can combat the development of heart failure in aging patients through the DSigDB database. The results show that Arsenenous acid, cyclophosphamide, lovastatin, Rimonabant hydrochloride, Sorafenib, and alvespimycin can interfere with some key aging genes. These drugs are mainly divided into anti-tumor drugs and lipid-lowering drugs. Arsenenous acid, cyclophosphamide, Sorafenib, and alvespimycin are predicted to be anti-tumor drugs, which may be related to key genes involved in cell cycle and programmed cell death. However, these anti-tumor drugs are generally cardiotoxic and pro-aging, which is a shortcoming of the DSigDB database (70, 71). Rimonabant, a cannabinoid receptor-1 (CB1) antagonist, shows promise in cardiovascular disease prevention (72). Research indicates that Rimonabant not only mitigates doxorubicin-induced cardiotoxicity but also effectively reduces inflammation and oxidative stress in the aging heart (73, 74). Furthermore, it combats aging-related insulin resistance and metabolic dysfunction, reverses obesity phenotypes in aged mice, and partially restores skeletal muscle function (75, 76). These findings suggest Rimonabant’s potential in delaying aging, enhancing metabolic health, and safeguarding cardiac function. Lovastatin, known as an HMG-CoA reductase inhibitor, is widely used clinically for cholesterol reduction and vascular atherosclerosis management. However, emerging studies reveal that beyond its cholesterol-lowering capabilities, lovastatin possesses anti-aging and anti-cancer properties (77, 78). Additionally, the dedifferentiating effects of statins may alleviate myocardial fibrosis in patients predisposed to heart failure (79). Consequently, the multifaceted mechanisms and therapeutic applications of statins like lovastatin in the realms of heart failure and aging warrant further exploration. These two medications may also have effects on immune cells. One study highlighted the anti-inflammatory action of Rimonabant on macrophages, which could imply a broader immunomodulatory effect, potentially influencing various macrophage states (80). Lovastatin has been proven to affect macrophages and T cells. It can influence the metabolism and function of macrophages (81). Additionally, lovastatin’s extensive anti-tumor activity may reduce the presence of immunosuppressive cells in the tumor microenvironment (82). The impact of these drugs on immune cells and aging merits further investigation. Although the use of machine learning combined with the DSigDB database for drug prediction does not ascertain the causal relationship between drugs and diseases, this comprehensive screening method substantially reduces the range of potential drugs. Therefore, this integrated machine learning approach has exciting application prospects and is worthy of further investigation.

Immune infiltration analysis showed that there were significant differences in the infiltration of two types of immune cells in aging myocardium. Macrophages M2 are a subpopulation of macrophages with anti-inflammatory effects. After receiving signals from IL-4 inflammatory factors, Macrophages M2 activate the secretion of anti-inflammatory cytokines such as IL-10 to inhibit M1 macrophages and promote wound healing and tissue repair (83).This could also be the reason why M1 macrophages are lower in elderly heart failure samples compared to those from younger heart failure patients. Impaired immune function associated with aging activates the innate immune system, systemic low-level chronic inflammation and the decline in the ability of macrophages to phagocytose pathogens can also lead to an increase in Macrophages M2 (84, 85). Higher levels of Macrophages M2 also reduce inflammatory damage to cardiomyocytes and delay fibrosis (86). This may be the reason why Macrophages M2 in elderly heart failure samples is higher than that in normal elderly samples. Studies have shown that depletion of CD8 + T lymphocytes reduces apoptosis in ischemic myocardium, hinders inflammatory responses, limits myocardial damage, and improves cardiac function (87). Lower T cell CD8 in elderly heart failure samples may be beneficial in maintaining cardiac function. Compared to younger patients with heart failure, myocardial samples from elderly patients exhibit lower expression of CD4 memory resting T cells and higher expression of regulatory T cells. This could be attributed to immunosenescence associated with aging, suggesting that these changes might not be exclusive to patients with heart failure (88, 89). In circulation levels, the resting NK cells of young heart failure patients are lower than those of elderly heart failure patients. This indicates that NK cells in younger patients are more active, while the activity of NK cells in older patients is relatively lower. This difference is primarily associated with age-related changes in the immune system. Therefore, this disparity may not be exclusive to heart failure patients (90).

The novelty of our research is as follows. First, we identified common genes in heart failure and aging through WGCNA and CellAge databases. Secondly, we identified key aging genes in heart failure through 3 machine learning algorithms. Notably, we fit 14 key genes on 15 machine learning algorithm models and 207 combinations and validated them in two independent external data sets. The results show that these 14 key genes can be used as aging signature genes in heart failure, which will help to further search for key aging-related mechanisms in the process of heart failure and develop specific drugs. Rimonabant and lovastatin, which we found through the DSigDB database, have the potential to delay aging and protect the heart. Finally, we evaluated the content of immune cells and stromal cells in myocardial samples from elderly patients with heart failure to provide potential regulatory points for the treatment of elderly heart failure.

Despite the contributions of this study, certain limitations must be acknowledged. Primarily, the correlation between the observed increases in mRNA levels and corresponding changes in protein expression remains uncertain. This is particularly relevant as the execution of numerous biological functions hinges on post-translational modifications. Secondly, despite employing 15 types of machine learning algorithm models and 207 combinations to validate the accuracy of key genes, it is still challenging to demonstrate a causal relationship between critical aging genes and age-related heart failure. If orthogonal methods were used, the cost of validating the causal link between 14 genes and the disease through animal experiments would be prohibitively high. Perhaps in the future, with a sufficient number of SNPs, we could employ Mendelian randomization to verify the causal relationship between them. Thirdly, while we have isolated genes that exhibit aging characteristics in heart failure, it is plausible that certain upregulated genes within heart failure serve as natural antagonists against cellular aging. Nevertheless, our research did not consider these genes. The paradoxical roles of these genes in heart failure and aging merit further investigation. Furthermore, there is a possibility that the CellAge and DSigDB databases might have overlooked some critical genes during their screening processes. In future research endeavors, should resources allow, we plan to incorporate experimental designs that assess protein levels and drug efficacy to substantiate and refine our conclusions more robustly.

## Conclusion

We performed bioinformatics analysis on the GEO dataset to explore the underlying molecular mechanisms and key genes of heart failure and aging. Through three machine learning algorithms: LASSO, RF and SVM-RFE, we identified 14 key aging genes in heart failure. After fitting 15 machine learning algorithm models and 207 combinations, and validating them in two independent external data sets, we determined that these 14 key genes can serve as aging signature genes for heart failure. Our exploration via the DSigDB database revealed rimonabant and lovastatin as promising agents capable of decelerating aging processes and offering cardiac protection. We also delineate the landscape of cellular heterogeneity in expression profiles of aging myocardium. Collectively, these insights pave the way for enhanced understanding of aging-related mechanisms in heart failure and could inform the development of targeted therapeutic interventions.

## Data availability statement

The original contributions presented in the study are included in the article/**Supplementary Material**. Further inquiries can be directed to the corresponding authors.

## Author contributions

YY: Conceptualization, Formal analysis, Investigation, Methodology, Resources, Validation, Visualization, Writing – original draft, Writing – review & editing. LW: Data curation, Software, Writing – review & editing. WH: Data curation, Software, Writing – review & editing. YX: Funding acquisition, Supervision, Writing – review & editing. XL: Funding acquisition, Project administration, Writing – review & editing. YL: Funding acquisition, Project administration, Writing – review & editing.
